# Is the Effect of a High-Intensity Functional Exercise Program on Functional Balance Influenced by Applicability and Motivation among Older People with Dementia in Nursing Homes?

**DOI:** 10.1007/s12603-019-1269-8

**Published:** 2019-10-04

**Authors:** Anna Sondell, H. Littbrand, H. Holmberg, N. Lindelöf, E. Rosendahl

**Affiliations:** 1 Department of Community Medicine and Rehabilitation, Physiotherapy, Umeå University, SE-90187 Umeå, Sweden; 2 Department of Community Medicine and Rehabilitation, Geriatric Medicine, Umeå University, Umeå, Sweden; 3 Department of Public Health and Clinical Medicine, Umeå University, Umeå, Sweden

**Keywords:** Dementia, exercise, postural balance, residential facilities

## Abstract

**Background and Objectives:**

Exercise can be an important way of maintaining balance function in people with dementia, but further investigation is needed to determine the optimal way of exercising. The objective was to evaluate whether exercise applicability (i.e., attendance, exercise intensity, and adverse events) and motivation were associated with the effect on functional balance of a high-intensity functional exercise program for older people with dementia in nursing homes.

**Design, Setting and Participants:**

Exercise intervention participants (n = 81; 60 women, 21 men) from a randomized controlled trial (UMDEX) were included. Their mean age was 84 and mean Mini-Mental State Examination score was 15.

**Intervention:**

Groups of 3–8 participants participated in the High-Intensity Functional Exercise (HIFE) Program, with 5 sessions per 2-week period, for 4 months (total, 40 sessions).

**Measurements:**

Outcome was the Berg Balance Scale (BBS), assessed at baseline and follow up, and the score difference, dichotomized to classify participants into two groups: responders (≥5-point increase) and non-responders (<5-point increase). Target variables were measures of applicability and motivation. Associations between each target variable and the outcome were analyzed using multivariable logistic regression. Baseline characteristics and new medical conditions developing during the intervention period were compared between responders and non-responders and included in the analyses when p < 0.10.

**Results:**

The BBS score was 28.6 ± 14.3 at baseline and 31.2 ± 15.3 at follow up, with the difference between follow-up and baseline scores ranging from −35 to 24. Twenty-nine (35.8%) participants were responders. The multivariable models showed no significant association between responders vs. non-responders and any target variable.

**Conclusion:**

Participation in a 4-month high-intensity functional exercise program can improve balance in many individuals with dementia in nursing homes, despite the progressiveness of dementia disorders and several co-existing medical conditions. Predicting balance exercise response based on applicability and motivation seem not to be possible, which lends no support for excluding this group from functional exercise, even when exercise intensity or motivation is not high.

## Introduction

Worldwide, dementia is common in older people and the leading cause of dependency in activities of daily living (ADL) (1). In addition to a gradual reduction in cognitive function, the negative consequences of dementia are multiple and complex, including balance and walking impairment (2). Such impairments contribute to increased risks of falls, fractures (3), physical inactivity (4), and dependency in ADL (5). People with dementia have about twice the risk of sustaining a fall as do people without dementia (6), and approximately 30% of older people who suffer hip fracture have dementia (7). Exercise seems to be an important way of maintaining balance function in people with dementia (8–10), but further investigation is needed to determine the optimal way of exercising for this group (8).

To improve functional ability and balance in older people, resistance (11, 12) and balance (13, 14) training is required. To achieve an optimal effect, the exercise should be task specific (15, 16) and performed at high intensity (near the individual’s maximum capacity) (3, 11, 16) and with sufficient frequency and duration (3, 12, 14). Concepts of motor learning may need to be considered in balance training to optimize transferability to daily life (17). Furthermore, an individual’s motivation to participate in exercise is important in maintaining an exercise program (18, 19), facilitating motor learning (17), and fulfilling exercise recommendations (12). However, exercise recommendations for older people are based mainly on findings from studies including older people without dementia (3, 11–14, 16).

In people with dementia, factors other than frequency, duration, intensity, and task-specify, and motivation may be important in achieving an exercise response. Due to reduced physiological reserve capacity, this population has a greater risk of sustaining other acute medical conditions than do people without dementia (20). Depression is common (21), as are symptoms such as anxiety, aggression, restlessness, hallucinations, wandering behavior, and sleep disturbances, which are referred to as behavioral and psychological symptoms of dementia (BPSD) (22). Other common symptoms that might impede exercise response include concentration difficulties, apraxia, perception disorder, fatigue, pain, and fear of falling. Furthermore, more than 70% of people with dementia exhibit apathy or lack of motivation or interest in activities (23, 24), which could affect their responsiveness to exercise. Additionally, people with dementia have a reduced ability to transfer when learning new skills and are, therefore, more dependent on implicit procedural learning. Hence, task specificity when exercising may be very important in this population (25). Consequently, combined consideration of these factors might influence the effect of exercise on balance and alter the associations of applicability (defined by the authors of this paper as attendance, intensity and presence of adverse events (26, 27)) and motivation with the exercise effect seen in older people in general. This potential difference might be especially true for people with dementia who reside in nursing homes, who form a heterogeneous group with high prevalence of comorbidities and medical conditions (20). However, the influence of these factors on the effect of exercise is sparsely studied in this population (28), and more research is needed to evaluate the importance of applicability and motivation for the achievement of positive balance effects (8).

The Umeå Dementia and Exercise (UMDEX) study evaluated the effects of a high-intensity functional exercise program in older people with dementia living in nursing homes. The majority of participants had high attendance rates and could exercise at moderate to high lower-limb strength intensity and high balance intensity, which led to only minor and temporary (mostly musculoskeletal) adverse events (27). The participants had high motivation levels in the majority of attended sessions, similar to those observed for the social control activity (29). The exercise group showed significantly improved balance in comparison with the control group after the intervention, but the effect differed according to dementia type. A positive effect was observed in participants with other types of dementia only in contrast to participants with Alzheimer’s disease (AD) (30). Participants with non-AD dementia performed more strength exercise sessions at high intensity than did participants with AD, which might explain the superior effect (27). However, whether factors such as attendance, intensity, adverse events and motivation are associated with the effect of exercise on functional balance in people with dementia living in nursing homes is not known.

### Aim

The aim of this study was to evaluate whether the applicability of exercise (in terms of attendance, exercise intensity, and adverse events) and motivation were associated with an effect on the functional balance outcome among older people with dementia living in nursing homes who participated in a high-intensity functional exercise program.

## Methods

This study was part of the UMDEX study, a cluster-randomized controlled trial conducted in 16 nursing homes in Umeå, Sweden. The nursing homes included general and dementia units; all had private rooms and staff on hand, as well as units with private apartments with access to on-site nursing and care. The UMDEX study has been described in detail elsewhere (30). The study protocol (ISRCTN31767087) is available on the ISRCTN registry. Ethical approval was obtained from the Regional Ethical Review Board in Umeå (2011-205-31M).

### Participants

Participants had dementia according to the DSM-IV-TR (31), ages ≥ 65 years, Mini-Mental State Examination (MMSE) scores ≥ 10 (32), dependence in personal ADL according to the Katz Index (score > A) (33), the ability to rise from a chair with an armrest with assistance from no more than one person, the ability to hear and understand spoken Swedish, and physicians’ approval to take part in the study. All participants provided informed oral consent, affirmed orally by next of kin. Those randomized to participate in the exercise intervention with determination of Berg Balance Scale (BBS) (34) scores at baseline and follow up were included in this study.

### Exercise Intervention

The intervention was based on the High-Intensity Functional Exercise (HIFE) program developed by research team members (26). This program, designed to improve lower-limb strength, balance, and mobility, is available on a webpage; http://www.hifeprogram.se/en (35). The HIFE program is composed of 39 exercises performed in functional weight-bearing positions, similar to those used in everyday situations (e.g., rising from a chair, trunk rotation, walking, climbing stairs). The exercises are distributed over five categories. The program recommendation is to perform at least two lower-limb strength exercises and two balance exercises in two sets each session (35). Exercise sessions were conducted at participating nursing homes in small groups (n = 3–8), each supervised by two physiotherapists (PTs). The sessions were led by PTs, who had experience in working with older people with dementia. Five sessions (~45 min each) were held per 2-week period for 4 months (total 40 sessions). Before the start of each exercise session, the PTs or nursing home staff members gave participants verbal reminders or aided their transfer to the exercise sessions, and, when needed, motivated participants to join the sessions. When possible, supervised individual sessions were provided for participants unable to attend group sessions. PTs were encouraged to obtain updates on participants’ health status before sessions and could contact physicians or nurses when necessary. PTs were able to adjust attendance arrangements and modify exercise intensity based on the participants’ health status.

Each group session started with seated group warmup exercises for all participants. Participants were then supervised individually to safely promote the highest possible exercise intensity. Participants took turns exercising and resting during each session. High intensity was the aim, with adaptation through progressive adjustment of load [performance adjustment or weighted waist belt (maximum 12 kg) use] and difficulty (e.g. narrowing base of support or surface alteration), while considering the participants’ symptoms and changes in health and functional status (35). PTs defined the intensity of each strength exercise set relative to the repetition maximum (RM), i.e. “the maximum number of times a load can be lifted before fatigue using good form and technique” (36) (high, 8–12 RM; medium, 13–15 RM; low, >15 RM). Balance exercise intensity, estimated by the supervising PTs through observation, was defined according to the level of postural stability challenge exhibited [high, fully challenged (i.e., balance exercises performed near the limit for maintaining an upright position); medium, not fully challenged or fully challenged in a minority of exercises; low, not challenged]. Participants wore belts with handles so that PTs could provide support when needed, thereby preventing falls. Participants performed moderate-intensity strength exercises for the first 2 weeks (build-up period) (35).

### Outcome variable

Functional balance was measured using the Berg Balance Scale (BBS). A participant’s ability to maintain an upright position while performing each of 14 tasks typical for everyday living (e.g., sitting, rising from sitting, transfer between two chairs, reaching while standing, standing with the eyes closed) was rated from 0 to 4, with independency as the highest score, and a maximum total score of 56 (34). Given that the ability to perform functional activities is multifaceted, the BBS also reflects aspects other than balance, such as lower-limb strength and dynamic movements, which are important for mobility. The BBS is a valid, reliable instrument for the measurement of balance function and evaluation of intervention effects, and it is recommended as a core outcome measure in clinical settings and for research in adult populations (37). Individual differences in BBS scores between follow-up and baseline testing were dichotomized to define two groups — responders (≥5 point increase) and non-responders (<5 point increase, including no change or decrease) — according to the minimal detectable change (MDC). A 5-point difference was selected in accordance with findings from an evaluation of intrarater test-retest reliability for BBS scores of people living in residential care facilities, which showed that a 5-point difference reflected a genuine change at an 80% confidence interval (38). PTs blinded to allocation and previous test results assessed balance at baseline and during follow up after the 4-month intervention [median, 15 days (range, 3–40 days after the intervention ended, with no difference between groups, p=0.83)].

### Target Variables

The target variables were attendance (number of sessions attended), intensity (number of sessions performed at high intensity for strength and balance exercises separately), adverse events occurrence (number of sessions in which adverse events occurred), and motivation (number of sessions performed with high or very high motivation). At the end of each exercise session, the PTs completed a structured protocol for each participant, which included the recording of exercise intensity (high, moderate, or low) (35), motivation during the exercise (5-point Likert scale: 0, none; 1, low; 2, moderate; 3, high; 4, very high) and adverse event occurrence, i.e., development or worsening of discomfort during the exercise session (observed by a leader or expressed by a participant spontaneously or upon questioning).

### Other Applicability Measures

The structured protocol also included the recording of the estimated effective workout time without rest. The PTs also assessed whether high-intensity strength exercises primarily strained the peripheral (muscular) or central (cardiorespiratory) systems. Through observation or questioning, they assessed whether participants’ primary reason for stopping exercise was lower-limb muscle fatigue or shortness of breath felt in the chest.

### Baseline Characteristics and New Medical Conditions during the Intervention

PTs and physicians performed baseline assessments before randomization. They assessed usual gait speed over 4 m with a walking aid, pain while walking (self-reported immediately after the gait speed test), depressive symptoms (through administration of the 15-item Geriatric Depression Scale during an interview) (39), global cognitive function (using the MMSE) (32), BPSD (according to the Neuropsychiatric Inventory, proxy-reported by nursing home staff) (40), nutritional status (using the Mini-Nutritional Assessment) (41) and ADL (using the Barthel ADL Index) (42). Barthel ADL Index item 7 was used to describe independence in walking, dichotomized as able or not able to walk independently. Diagnoses were based on information gathered from assessments, medical records, and medication prescriptions. Physicians specialized in geriatric medicine diagnosed dementia types according to the DSM-IV-TR (31).

New medical diagnoses and hospital stays during the intervention period were followed through review of participants’ medical records. Falls during the intervention were followed through medical records and fall-incidence reports filed at the nursing homes. Medical events and illness during the intervention, which may have influenced applicability, motivation or exercise effects, but did not require hospitalization, were merged into a single dichotomous variable (including stroke or other symptoms from the central nervous system, mental illness, heart and lung diseases, diabetes, osteoarthritis, fractures, infections, and malignancy).

### Data Analysis

Baseline characteristics of the participants, target variables, other applicability data obtained during the intervention, new medical diagnoses, and data on falls and hospital stays during the intervention were compared between responders and non-responders using univariate logistic regression. Variables showing significant differences (p < 0.10) between groups in this univariate analysis were included in multivariate logistic regression models with responder/non-responder serving as the dependent variable. Independent variables included in the models were attendance, MMSE score, Barthel ADL Index item 7 score, and one target variable (attendance, high intensity in strength and balance exercises, adverse events, or high motivation) per model. All models were adjusted for attendance (except the first model, which included attendance) because the variables were related to overall attendance. Previous hip fracture was significant at the p < 0.10 level in the univariate logistic regression analysis, but was excluded due to correlation with the Barthel ADL Index item 7 score (r = 0.328) and interference with that variable in the regression models. The Barthel ADL Index was not entered in the models because item 7 was chosen as a measure of independence in walking, and because it showed strong correlation with the BBS score (r = 0.685) and correlation with the MMSE score (r = 0.348). Peripheral strain was not entered in the model that included high intensity in strength exercises due to strong correlation between these variables (r = 0.868). Correlation between all variables in the multivariate models was tested using the Spearman rank correlation coefficient, and no other strong correlation (r > 0.65) was found.

Additionally, multivariable linear regression analysis was performed with the difference in BBS scores between follow up and baseline serving as a continuous dependent variable. The independent variables were the same as in the multivariate logistic regression models. This analysis was performed to evaluate whether the cutoff value for the BBS score used in the logistic regression models would lead to misleading results when equalizing decreasing score change with increasing score change below the cutoff value. All analyses were performed using IBM SPSS (version 23) and R Core Team (2017) (43) software, with a two-tailed significance level of p < 0.05.

## Results

**Figure 1:**
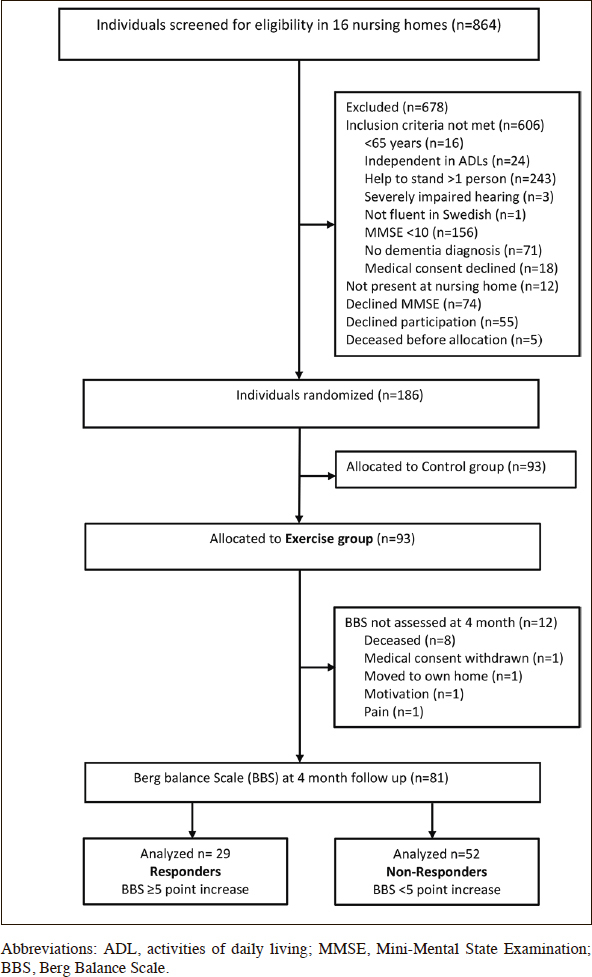
Flowchart of participants

Eighty-one participants (60 women, 21 men) were included in the study (Figure 1). Thirty (37%) participants had AD and 51 (63%) had non-Alzheimer’s types of dementia, including vascular, mixed Alzheimer’s and vascular, Lewy body, frontotemporal, and Parkinson’s dementias. The participants’ mean ± standard deviation (SD) MMSE score was 15.4 ± 3.4. More than half (59.3%) of the participants had depressive disorder, 45.7% had delirium in the preceding week, and one-third (33.1%) had suffered stroke. Three of every 10 (29.6%) participants had sustained previous hip fractures, and four of every five (81.5%) used mobility devices, such as wheelchairs and walkers (Table 1). The median [interquartile range (IQR)] number of attended sessions was 34 (30–37). The participants performed high-intensity strength exercises at a median (IQR) of 16 (3.5–27.0) sessions and high-intensity balance exercises at a median (IQR) of 24 (11.0–31.5) sessions. The median (IQR) effective workout time per session was 17.6 (15.1–19.6) min. During the intervention period, nearly half (44.4%) of the participants fell, 12.3% were admitted to the hospital, and almost half (45.7%) developed medical events and illness without hospitalization (Table 2).

**Table 1:** Baseline Characteristics of Participants

**Characteristic**	**Total (n=81)**	**Responders BBS ≥5 increase (n=29)**	**Non-responders BBS <5 increase (n=52)**	**OR (95 %CI)**	**P value**
Age, years	84.1±6.2	84.5±7.1	83.8±5.7	1.02 (0.95–1.10)	0.636
Sex, female	60 (74.1)	22 (75.9)	38 (73.1)	1.16 (0.41–3.30)	0.784
*Dementia type*					
AD	30 (37.0)	10 (34.5)	20 (38.5)	0.84 (0.33–2.17)	0.722a
Non-AD	51 (63.0)	19 (65.5)	32 (61.5)		
Vascular	31 (38.3)	13 (44.8)	18 (34.6)		
Mixed-AD/vascular	6 (7.4)	1 (3.4)	5 (9.6)		
Other	14 (17.3)	5 (17.3)	9 (17.3)		
*Diagnoses and medical conditions*					
Depressive disorders	48 (59.3)	16 (55.2)	32 (61.5)	0.77 (0.31–1.93)	0.576
Delirium, previous weekj	42 (51.9)	13 (44.8)	29 (55.8)	0.64 (0.26–1.61)	0.346
Previous stroke	27 (33.1)	12 (41.4)	15 (28.8)	1.74 (0.67–4.51)	0.253
Heart failure	21 (25.9)	9 (31.0)	12(23.1)	1.50 (0.54–4.15)	0.435
Angina pectoris	17 (21.0)	9 (31.0)	8 (15.4)	2.48 (0.83–7.36)	0.103
Previous hip fracture	24 (29.6)	5 (17.2)	19 (36.5)	0.36 (0.12–1.11)	0.074
Rheumatic disease	13 (16.0)	6 (20.7)	7 (13.5)	1.68 (0.51–5.57)	0.399
Chronic lung disease	18 (22.2)	8 (27.6)	10 (19.2)	1.60 (0.55–4.65)	0.388
Osteoarthritis	32 (39.5)	11 (37.9)	21 (40.4)	0.90 (0.36–2.29)	0.829
Hearing impairment	17 (21.0)	7 (24.1)	10 (19.2)	1.34 (0.45–4.00)	0.604
Vision impairment	9 (11.1)	2 (6.9)	7 (13.5)	0.74 (0.37–1.47)	0.392
Pain while walking	11 (13.6)	4 (13.8)	7 (13.5)	0.99 (0.76–1.30)	0.935
*Prescription medications*					
Analgesics	48 (59.3)	17 (58.6)	31 (59.6)	0.96 (0.38–2.42)	0.930
Antidepressants	50 (61.7)	16 (55.2)	34 (65.4)	0.65 (0.26–1.65)	0.366
Benzodiazepine	15 (18.5)	3 (10.3)	12(23.1)	0.53 (0.16–1.84)	0.319
Diuretics	34 (42.0)	12 (41.4)	22 (42.3)	0.96 (0.38–1.42)	0.935
Anti-dementia drugs	25 (30.9)	6 (20.7)	19 (36.5)	0.45 (0.16–1.31)	0.144
Neuroleptics	10 (12.3)	3 (10.3)	7(13.5)	0.74 (0.18–3.12)	0.683
Number of medications	8.3±3.9	8.5±4.7	8.2±3.4	1.02 (0.91–1.15)	0.700
*Assessments*					
Barthel ADL Index (0-20)§	10.8±4.6	12.3±4.0	10.0±4.7	1.13 (1.01–1.26)	0.034*
Barthel ADL index, item 7; able to walk independently	44 (54.3)	20 (69.0)	24 (46.2)	2.59 (1.00–6.75)	0.051
MMSE (range 0-30)§	15.4±3.5	16.3±3.4	14.9±3.5	1.13 (0.99–1.29)	0.075
BBS (range 0-56)§	28.8±14.0	27.9±11.6	29.3±15.2	0.99 (0.96–1.03)	0.654
Gait speed 4 m, m/s	0.48±0.4	0.47±0.2	0.48±0.2	0.69 (0.07–7.24)	0.758
NPI (range 0-144)11	15.9±16.2	16.4±16.4	15.6±16.3	1.00 (0.98–1.03)	0.823
GDS-15 (range 0-15)11	4.0±3.3	4.5±3.8	3.6±2.9	1.09 (0.95–1.25)	0.244
MNA (range 0-30)§	21.3±2.7	21.5±2.7	21.2±2.7	1.04 (0.87–1.23)	0.690
Use of mobility device	66 (81.5)	24 (82.8)	42 (80.8)	1.02 (0.79–1.31)	0.901
Self-reported health, good	52 (64.2)	18 (62.1)	34 (65.4)	0.87 (0.34–2.22)	0.765
Life-space, daily transfer out of the ward	23 (28.4)	6 (20.7)	17 (32.7)	0.54 (0.18–1.56)	0.254

Values are expressed as mean ± standard deviation or n (%); *α* Difference between AD and Non-AD; * Significant (p<0.05); †Reported by staff based on the confusion subscales of the Organic Brain Syndrome Scale; §Higher scores indicate better status; IILower scores indicate better status; Abbreviations: BBS, Berg Balance Scale; BBS ≥5 increase, difference between follow up and baseline ≥5; BBS < 5 increase, difference between follow up and baseline <5; AD, Alzheimer’s disease; ADL, activities of daily living; MMSE, Mini-Mental State Examination; NPI, Neuropsychiatric Inventory; GDS-15, 15-item Geriatric Depression Scale; MNA, Mini Nutritional Assessment.

**Table 2:** Intervention related measures

	**Total (n=81)**	**Responders BBS ≥5 increase (n=29)**	**Non-responders BBS <5 increase (n=52)**	**OR, 95% CI**	**P value**
*Target variables; median (IQR)*					
Attendance, n	34.0 (30.0–37.0)	34.0 (29.0–37.0)	35.0 (30.3–38.0)	0.98 (0.94–1.03)	0.418
High Intensity strength, n	16.0 (3.5–27.0)	15.0 (3.0–23.5)	21.0 (6.5–27.8)	0.97 (0.93–1.01)	0.167
High Intensity balance, n	24.0 (11.0–31.5)	21.0 (9.0–28)	24.0 (14.3–33.0)	0.97 (0.93–1.01)	0.116
Adverse event, n	2.0 (0.5–6.0)	3.0 (0–9.5)	2.0 (1.0–5.0)	1.02 (0.96–1.10)	0.498
High Motivation, n	20.0 (8.0–30.0)	10.0 (4.5–28.5)	23.0 (9.5–31.0)	0.97 (0.94–1.01)	0.158
*Other applicability variables; median (IQR)*					
HI strength + balance, n	13.0 (3.0–25.0)	9 (3.0–22.8)	19.0 (5.0–26.0)	0.97 (0.93–1.02)	0.217
HI+MI strength, n	32 (23.0–36.0)	30.0 (20.0–34.0)	33.0 (24.3–36.8)	0.97 (0.93–1.01)	0.172
HI+MI balance, n	33 (27.5–37.0)	31 (26.0–35.5)	33.5 (28.3–37.0)	0.98 (0.94–1.02)	0.319
Effective workout time/session minutes	17.6 (15.1–19.6)	16.6 (14.1–19.3)	17.9 (15.3–19.7)	1.00 (1.00–1.00)	0.362
Peripheral strain*, n	13.0 (5.5–23.5)	8.5 (2.5–22.5)	19 (8.5–25.0)	0.96 (0.91–1.01)	0.075
*New medical conditions †, n (%)*					
Falls	36 (44.4)	13 (44.8)	23 (44.2)	1.02 (0.41–2.56)	0.959
Hospital stay	10 (12.3)	5 (17.2)	5 (9.6)	1.52 (0.62–3.73)	0.357
Medical event and illness without hospitalization	37 (45.7)	11 (37.9)	26 (50.0)	0.61 (0.24–1.54)	0.297

Values are expressed as median (IQR) or n (%); *Number of sessions where muscle fatigue in the lower-limb were the reason for stopping, in sessions with high intensity in strength exercises; † during the intervention; Abbreviations: BBS, Berg Balance Scale; BBS ≥5 increase, difference between follow up and baseline ≥5; BBS <5 increase, difference between follow up and baseline <5; HI, High Intensity; HI+MI, High Intensity + Moderate Intensity.

**Figure 2:**
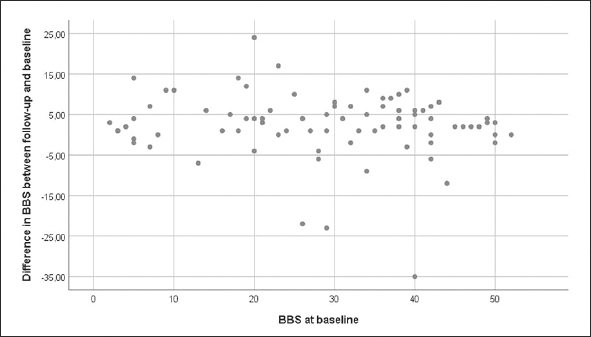
Association between difference in BBS between follow up and baseline and BBS at baseline. Positive value on y-axis indicates an increase, and a negative value indicates a decrease. BBS, Berg Balance Scale

**Figure 3:**
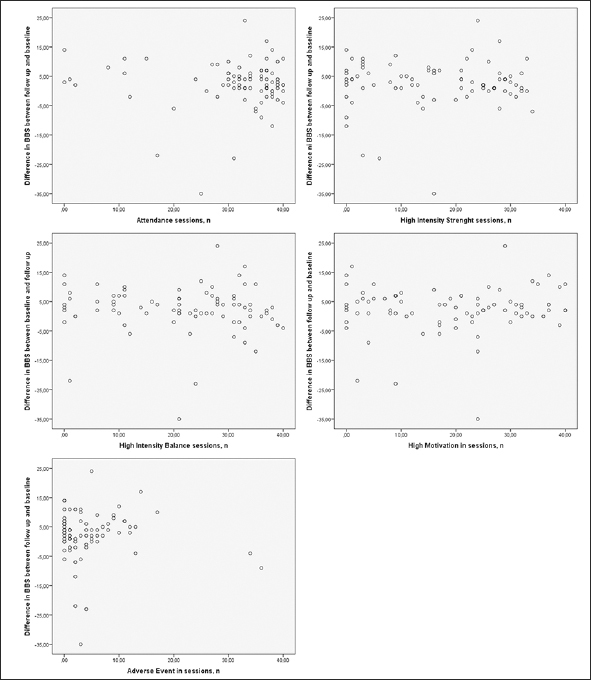
A-E Associations between difference in BBS between follow up and baseline and the target variables. BBS, Berg Balance Scale

The mean ± SD BBS score was 28.6 ± 14.3 at baseline and 31.2 ± 15.3 at follow up. The range of difference between BBS follow-up and baseline scores was −35 to 24 points: scores of 17 participants decreased, 5 were unchanged, and 59 increased. Univariate associations between the differences in BBS scores and the baseline BBS score are shown in Figure 2, and those between the differences in BBS scores and target variables are shown in Figure 3. Twenty-nine (35.8%) participants were classified as responders (≥5 point increase) and 52 (64.2%) participants were classified as non-responders (<5 point increase). The mean BBS score difference ± SD (range) for the responders was 9.1 ± 4.2 (5–24), and that for the non-responders was −1.3 ± 7.8 (−35–4). Data on baseline characteristics, including odds ratios (ORs) for responders vs. non-responders, are shown in Table 1. The only significant difference was in the Barthel ADL Index, which was higher among responders (12.3 ± 7.1 vs. 10.0 ± 4.7, p = 0.034). No target variable, other applicability measure, or new medical condition during the intervention differed significantly between responders and non-responders in the univariate regression analysis (Table 2).

No significant association was found in any multivariate model between responders vs. non-responders and a target variable or adjusting variable (Table 3). The additional multivariate linear regression models revealed no significant association between differences in BBS scores and a target variable or adjusting variable (data not shown).

**Table 3:** Multivariable logistic regression models analyzing association between for Berg Balance Scale responders and non-responders, target variables and adjusting variables

**Model**	**Target Variable**	**OR**	**95 % CI**	**P value**
1	Attendance†	0.996	0.986–1.007	0.510
2	High Intensity Strenght♯	0.992	0.981–1.003	0.149
3	High Intensity Balance♯	0.989	0.977–1.001	0.089
4	Adverse event♯	1.009	0.992–1.026	0.329
5	High Motivation♯	0.992	0.982–1.002	0.104

Multivariable logistic regression models (1–5) with responders/non-responders as a dependent variable. Independent variables were attendance, MMSE and Barthel ADL index item 7 and a target variable; † number of sessions; ♯ number of attended sessions with target variable.

## Discussion

In this study, which involved a 4-month high-intensity functional exercise program intervention for older people with dementia living in nursing homes, a large degree of individual variance in the difference between follow-up and baseline functional balance was found. Many participants improved their functional balance, despite having progressive dementia and, in many instances, other medical conditions. The applicability of the exercise program (in terms of attendance, exercise intensity, and the occurrence of adverse events), as well as, motivation during exercise, did not differ significantly between responders and non-responders.

To our knowledge, no previous study has evaluated the association of exercise effect with motivation, exercise intensity, or the occurrence of adverse events in people with dementia. The association between attendance and exercise effect has been evaluated in two studies (28, 44). A study conducted in Norway (28) revealed such an association in nursing home residents who participated in the HIFE program; an attendance rate of ≥50% was associated significantly with improvement in the chair-stand test, but not with improvement in the BBS score. The chair-stand test does not measure balance alone; it is a functional measure and chair stand ability is included in the BBS. However, direct comparison of this finding with our results is not possible, as that study included a control group in the analysis, and the participants had fewer comorbidities and better physical function than did our population (28). In line with our findings, a study conducted in Germany (44) showed that attendance was not a significant predictor of successful training response for the chair-stand test. That study was conducted in outpatient facilities, and most participants were community-dwelling older people with dementia who had higher levels of cognitive and physical function and fewer comorbidities than did our population (44). In addition, comparison of our results with those of systematic reviews of the effects of balance interventions that included older participants, nearly all of whom were community dwelling and without dementia, yielded inconclusive findings (3, 14, 45). The authors of one review concluded, in line with our results, that exercise program modalities inadequately explained the balance effect (45). In contrast, a review of balance exercise in healthy older adults identified effective balance training protocol comprising exercise modalities (duration, frequency) (14). Exercise intensity was not evaluated in those reviews because it was not reported in the included studies. This factor has previously been noted, with a call for further research (46). Lastly, a review of exercise regimens aiming to prevent falls showed that exercise programs that challenge balance and include higher doses of exercise had greater effects on falls prevention (3). Given the inconclusive nature of reported findings, more research exploring how exercise modalities affect balance exercise responses in various groups of older people, including those with dementia, is needed.

The lack of association between exercise effect and applicability and motivation in this study represents a complex matter with several possible explanations. People with dementia who live in nursing homes form a heterogeneous group with progressive disease along with comorbidities and additional new medical conditions (20), also seen in our study, that can confound and reduce responsiveness to exercise. We found, however, no difference between responders and non-responders in baseline characteristics or the development of new medical conditions during the intervention in the univariate analysis, with the exception of greater ADL function among responders. Furthermore, participants’ physical activity levels and ability in daily life might have influenced the effect of exercise on functional balance observed in this study. Most people with dementia living in nursing homes are inactive and spend their days inactive, in lying or sitting positions, in the ward (47). Some participants with low physical activity levels might have benefitted simply from the extra physical activity that they performed during the present intervention, which included transfer to the exercise session locations in the nursing home facilities and exercise (regardless of intensity) during the sessions. In the UMDEX study, the control group performed a seated activity at the same frequency and duration, which also included transfer to the activity location (30). BBS scores decreased by a mean of 1.8 points in this group compared with a mean increase of 2.3 points in the exercise group, but with a large degree of individual variability (range, −25 to 21). Additionally, discrepancies may have existed between participants’ physical capacity (what they could do) and their physical performance in daily life (what they actually did) (48). People living in nursing homes might not use their full physical capacity in daily life, as they may receive help with care even when they are able to do things themselves48. Additional reasons for not using their full physical capacity might be related to the need for support and lack of opportunity, intention, and suitable activities (49), which would affect the exercise response negatively. Hypothetically, even if the participants who exercised at high intensity and had high attendance rates achieved the most improvement during exercise sessions, the lasting effects might have been blurred by their non-use of this improved capacity in daily life. The importance of being able to use one’s own capabilities, without support from others, in achieving a lasting exercise effect is reflected by responders’ higher Barthel ADL Index scores and the greater proportion tendency of participants who walked independently (as reflected by univariate regression results) in the present study.

The majority of participants in our study had high attendance rates, and could perform strength exercises at moderate to high intensity and balance exercises at high intensity, in accordance with exercise recommendations. Despite meeting these recommendations, however, the functional balance effect showed a large degree of individual variance. Such pronounced variance from group means has also been found in other exercise studies conducted with older people without dementia (50–52). Individuals can respond differently to the same type of exercise and the identification of factors that influence individual exercise responses among older people can have great clinical significance (50). Factors proposed to be associated with exercise response include hereditary factors, the pre-training phenotype, characteristics of the exercise program (intensity, frequency, duration), activity level, functional level, lifestyle factors, recovery and sleep between sessions, dietary intake, and measurement-associated factors (53). For functional measures, an exercise response can also be obtained at lower exercise intensity and frequency; for example, in a meta-analysis of resistance training in older people, the effect was independent of exercise intensity (54).

Some group differences that were contrary to expectations based on exercise recommendations for older people were found, although these were not statistically significant. Responders participated in fewer sessions with high motivation and high intensity in strength and balance exercises, and more sessions with adverse events, than did non-responders. A possible explanation for these findings is that responders had non-significantly larger proportions of heart and lung disease and stroke, the symptoms of which can affect the applicability of exercise. They also performed fewer sessions with peripheral strain, which entails a greater proportion of central loading in high-intensity strength exercises. Because of the larger proportions of heart and lung disease and stroke, this group might have been more inactive before the intervention, and thus shown a greater exercise effect despite the lower intensity of exercise (55, 56). The presence of neurological disease was associated with a greater response, as reflected by BBS scores, in the study conducted in Norway (28), which might be in line with the larger proportion of stroke survivors among responders in our study. Responders also had non-significantly greater MMSE score, which might have affected the exercise response. Overall, these results, although not significant, support the interpretation that the associations between exercise and its effects in people with dementia living in nursing homes are complex.

Our results show that the prediction of who will respond to balance exercises can be difficult. The fact that balance is a multifaceted and complex function can contribute to these findings. Balance training must consider concepts of motor learning (including interaction among the individual, the task, and the environment, as well as practice method) (17). Reduced motor learning ability due to neurodegenerative disease and cognitive impairment might also influence the effect of exercise in people with dementia (17, 25). Furthermore, individuals with dementia have a reduced ability to transfer when learning new skills and are more dependent on implicit procedural learning. Hence, consideration of task specificity may be more important in designing exercise programs for this population than in designing exercise programs for older people in general (25). However, we believe that exercise should be prescribed for people with dementia according to exercise recommendations regarding exercise intensity and frequency. We assert this despite the lack of support of the present findings for the importance of exercise prescription. Emerging evidence shows positive exercise effects when exercise recommendations are followed (8, 28, 30, 57). Future studies are needed to determine which exercise modalities and individual characteristics are important for optimal exercise responses in this population.

Some limitations of this study should be considered. This study may have lacked statistical power due to the small group sizes. In addition, the results should be interpreted with caution, as exercise modalities (e.g., frequency and intensity) were not randomized, and were analyzed as actually performed. Another consideration is whether the selected measure (the BBS) actually captured the effect of exercise for all participants. A review, however, confirmed the suggested use of the BBS as a core measure of functional balance outcomes in adults (37). Furthermore, the dichotomization of outcome variables has disadvantages; data variety is lost, and participants with deteriorated balance are equalized with those with balance improvement below the MDC. However, we found no significant associations when using the total difference in BBS scores as a continuous dependent variable. It might be difficult to relate the MDC in a population with a progressive disease in which the preservation of function can be considered a positive result, as nursing residents shown as an average of 2% decrease in functional balance according to BBS per month58. A <5-point increase in a group with a progressive disease might still have an effect on everyday life over time. Another limitation of the study is that we did not assess each participant’s overall physical activity at baseline or during the intervention. The life-space assessment performed at baseline determined how often participants left their wards, but not how they did so (e.g., by walking independently or being pushed in a wheelchair). Furthermore, the medical event and illness without hospitalization variable was generated by gross fusion of multiple new medical conditions that emerged during the intervention. This single variable might not have captured all relevant data. Additionally, PTs subjectively estimated exercise intensity and participants’ motivation during exercise, which weakens the reliability of the assessments. However, the rating system was standardized, and all PTs were familiar with it in advance. Finally, generalization of the findings is limited to people with dementia who live in nursing homes and exercise in a similar way. However, the study population showed broad variation in physical and cognitive capacities. One strength of the study is the broad inclusion of factors that may affect the exercise response. Another strength is the collection of extensive data on applicability and motivation. Additionally, the intensity scales for balance exercises included in the HIFE program were well defined, as called for in a previous study (46).

## Conclusion

Despite individual variation, participation in a 4-month high-intensity functional exercise program may improve balance in many individuals with dementia living in nursing homes. Such improvement can be observed despite the progressiveness of dementia disorders and several co-existing medical conditions. High attendance, exercise intensity, and motivation rates, as well as the occurrence of few adverse events during the exercise program, may not be associated with paramount balance response. The prediction of balance exercise response based on applicability and motivation does not seem to be possible, which lends no support to the exclusion of this group from functional exercise, even when exercise intensity or motivation to exercise is not high.

## Notes

*Ethical Standards:* This study complies with current laws on research in Sweden.
